# Retraction: A Deep Neural Network for Estimating Low-Density Lipoprotein Cholesterol From Electronic Health Records: Real-Time Routine Clinical Application

**DOI:** 10.2196/108367

**Published:** 2026-08-19

**Authors:** JMIR Publications Editorial Office

**Affiliations:** 1 JMIR Publications Toronto, ON

The JMIR Publications Editorial Office is retracting the article “A Deep Neural Network for Estimating Low-Density Lipoprotein Cholesterol From Electronic Health Records: Real-Time Routine Clinical Application” [1] due to concerns regarding third-party involvement in the submission and the integrity of the peer review process.

The authors stated that the absence of disclosure regarding a previous copublication with reviewer T Lee in accordance with JMIR Publications policy was an inadvertent omission.

However, the Editorial Office and editor in chief determined that there is sufficient evidence indicating that both the submission process and peer review process for this article were compromised.

The authors did not respond to the decision to retract the article.
